# C/EBP-δ positively regulates MDSC expansion and endothelial VEGFR2 expression in tumor development

**DOI:** 10.18632/oncotarget.16410

**Published:** 2017-03-21

**Authors:** Yongfen Min, Jingdong Li, Peng Qu, P. Charles Lin

**Affiliations:** ^1^ Center for Cancer Research, National Cancer Institutes, Frederick, MD 21702, USA; ^2^ Department of Hepatobiliary Surgery, Affiliated Hospital of North Sichuan Medical College, Nanchong 637007, Sichuan, China; ^3^ Hepatobiliary, Pancreatic and Intestinal Diseases Research Institute, North Sichuan Medical College, Nanchong 637007, Sichuan, China

**Keywords:** MDSCs, C/EBP-δ, VEGFR2, angiogenesis, cancer

## Abstract

Vascular endothelial cells and Gr-1+CD11b+ myeloid derived suppressor cells (MDSCs) are two important components that constitute the tumor microenvironment. Targeting these cells offers the potential to halt tumor growth. In this study, we report a common mediator in C/EBP-δ that regulates both components and aids in tumor development. C/EBP-δ is elevated in tumor derived MDSCs. Interestingly, genetic deletion of C/EBP-δ in mice significantly impaired MDSC expansion in response to tumor progression, but it had no effect on Gr-1+CD11b+ cell production in normal development. It suggests a specific role of C/EBP-δ in emergency myelopoiesis under tumor conditions. Consistent with the pro tumor functions of MDSCs, loss of C/EBP-δ resulted in reduced tumor angiogenesis and tumor growth. Moreover, we found expression of C/EBP-δ in vascular endothelial cells. C/EBP-δ regulated cell motility, endothelial network formation and vascular sprouting. Notably, inactivation of C/EBP-δ in endothelial cells specifically inhibited the expression of VEGFR2 but not VEGFR1. Ectopic expression of C/EBP-δ increased and knockdown of the gene decreased VEGFR2 expression. C/EBP-δ is recruited to the promoter region of VEGFR2, indicative of transcriptional regulation. Collectively, this study has identified a positive mediator in C/EBP-δ, which regulates tumor induced MDSC expansion and VEGFR2 expression in endothelium. Considering the importance of MDSCs and endothelial cells in tumor progression, targeting C/EBP-δ may provide an interesting means for cancer therapy, killing two birds with one stone.

## INTRODUCTION

Tumor growth and progression depends on tumor angiogenesis, which is a multi-step process requiring sequential activation of different angiogenic pathways. Among them, vascular endothelial growth factor (VEGF) signaling plays a pivotal role. Multiple VEGF isoforms have been identified with VEGF (VEGF-A) as a major factor in angiogenesis mainly through interaction with VEGF receptor 2 (VEGFR2) on vascular endothelium [6]. Activation of the VEGF/VEGFR2 axis triggers multiple signaling networks that result in cell proliferation, migration, assembly into vascular networks, and endothelial cell survival.

Myeloid derived suppressor cells (MDSCs, Gr-1+CD11b+) play important roles in tumor growth and progression. These cells are also called immature myeloid cells and myeloid immune suppressor cells. This population of cells is dramatically elevated in cancer patients and tumor bearing animal models [19]. They inhibit immune cell functions and compromise host immune surveillance [8, 18]. In addition, these myeloid cells infiltrate into tumors, modulate the tumor microenvironment and directly promote tumor angiogenesis and tumor growth through regulating the bioavailability of VEGF. MDSCs also promote tumor cell invasion and metastasis via regulating protease activities in tumor tissues [30, 31]. Moreover, the angiogenic properties of MDSCs in tumor-bearing mice contribute to tumor resistance to anti-VEGF treatment [25]. Despite the importance of these myeloid derived suppressor cells in cancer growth and progression, the mechanisms regulating the expansion of these cells in tumor conditions are less clear.

C/EBP-δ belongs to the C/EBP family, which consists of a group of six structurally and functionally related transcription factors (C/EBP-α,-β,-δ,-ε,-γ, and -ζ) [15, 33]. C/EBP-δ is strongly induced by inflammatory cytokines [22, 27]. Consistent with the close association of inflammation to cancer development, the level of C/EBP-δ in carcinoma is significantly higher than surrounding normal tissue [13, 20], indicating a positive role of the gene in tumorigenesis. Indeed, we recently reported that C/EBP-δ regulates lymphangiogenesis and tumor metastasis [21]. C/EBP-δ is known to regulate cell differentiation, which includes myeloid lineage cells [9, 24], neutrophil [3], and myeloid leukemia cells [10]. In addition, C/EBP-δ regulates gene expression, including VEGFR3 in lymphatic endothelial cells [21], platelet-derived growth factor receptor (PDGFR) in vascular smooth muscle cells [7, 14, 32], insulin-like growth factor-I in osteoblast cells [4, 11], CXCR4 and HIF-1α in tumor cells and macrophages [1, 2].

In this study, we identify C/EBP-δ as a common mediator that regulates the expansion of MDSCs in tumor conditions as well as VEGFR2 expression in vascular endothelial cells, two important populations of cells that constitute the tumor microenvironment. Loss of this transcription factor impairs the expansion and tumor infiltration of MDSCs as well as VEGFR2 expression in endothelium that together resulted in a reduction of tumor angiogenesis and tumor growth. These findings suggest C/EBP-δ as a potential therapeutic target for cancer therapy. It should simultaneously target MDSCs and tumor endothelium, two important components in the tumor microenvironment, to achieve killing two birds with one stone.

## RESULTS

### C/EBP-δ regulates the expansion of MDSCs in tumor conditions

**Figure 1:**
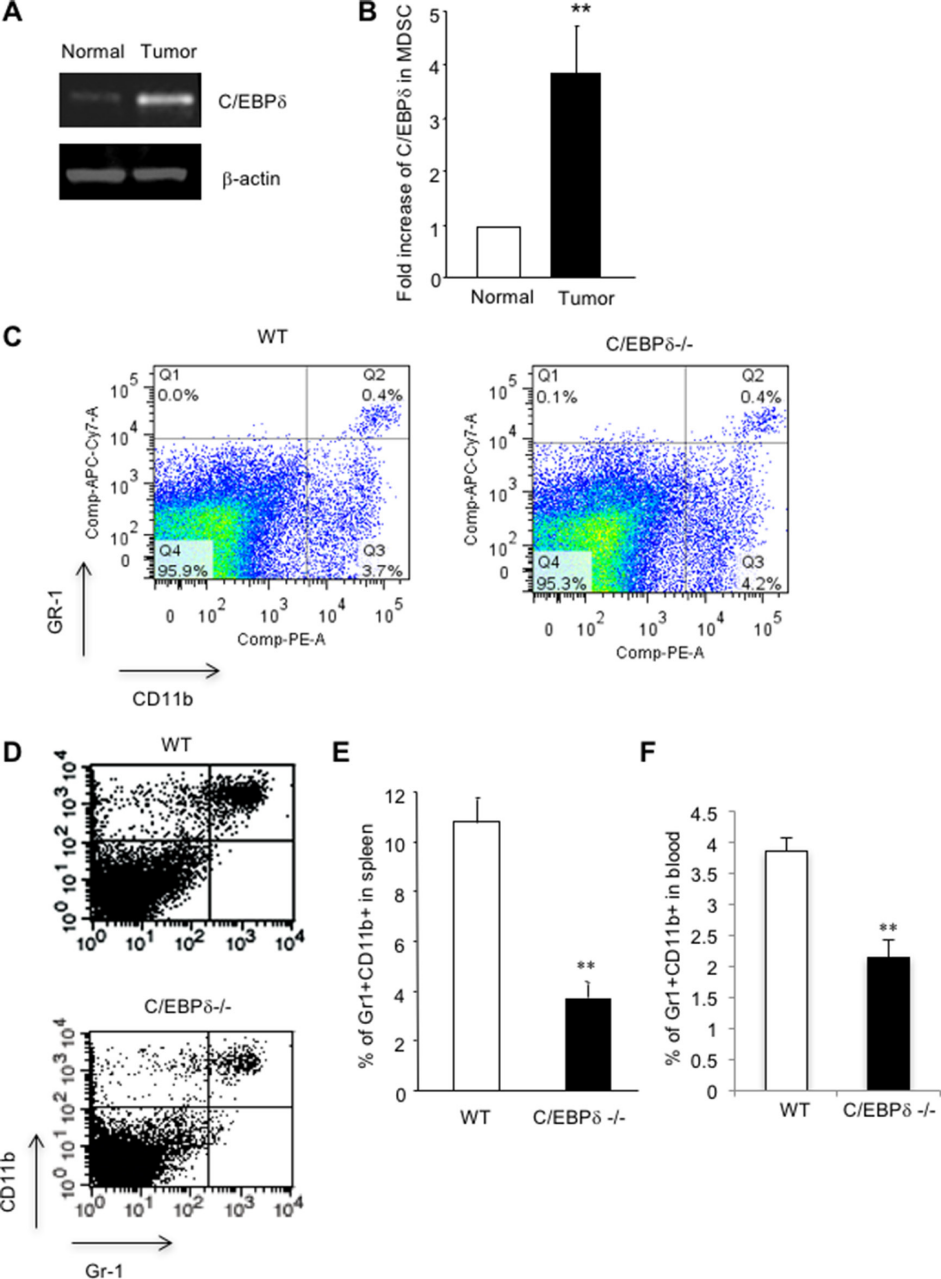
C/EBP-δ regulates tumor-induced expansion of MDSCs Gr-1 and CD11b double positive cells were isolated by FACS sorting from spleens of C57Bl/6 mice with or without subcutaneous (s.c.) implantation of 3LL tumors. Total RNA was isolated and subjected to semi-quantitative RT-PCR (Panel **A**) and qPCR (Panel **B**) for C/EBP-δ expression. *n* = 3 mice per group, **p < 0.01. Gr-1CD11b double positive cells were analyzed by flow cytometry in spleens from age and sex matched C/EBP-δ null mice and littermate WT mice (Panel **C**) *n* = 5 mice per group. 5 × 10^5^ 3LL tumor cells were s.c. implanted into age and sex matched C/EBP-δ null mice and littermate WT mice for approximately 3 weeks. Single cell suspensions were made from spleens of mice with similar size tumors and analyzed by flow cytometry for Gr-1 and CD11b double positive cells (Panel **D**). The levels of Gr-1+CD11b+ cells in spleens (Panel **E**) and blood (Panel **F**) of tumor-bearing mice were plotted. *n* = 7 mice per group, **p < 0.01. Each experiment was repeated twice. Representative flow images are shown.

MDSCs are myeloid cells that are dramatically increased in cancer patients and tumor bearing animal models. Murine MDSCs express Gr-1 and CD11b cell surface markers, which are then used to mark this population of cells in mouse models. Despite the significance of these myeloid cells in tumor growth and progression, what regulates the expansion of MDSCs in tumor conditions is less clear. In searching for potential mediators, we compared gene expression between Gr-1+CD11b+ cells derived from tumor-bearing mice and non-tumor bearing mice. We found that C/EBP-δ was significantly increased in tumor host derived MDSCs compared to non-tumor bearing hosts (Figure 1A and 1B), which suggests a positive role of C/EBP-δ in MDSC development.

As C/EBP-δ is implicated in myeloid cell differentiation [9, 24], we reasoned a role of this transcription factor in the expansion of MDSC in tumor conditions. To test this hypothesis, we used C/EBP-δ null mice. Mice without the C/EBP-δ gene are viable, grossly normal and fertile except subtle defects in adipocyte differentiation, mammary gland involution, and specific types of learning and memory [12, 26, 28]. Initially, we harvested spleens from age and sex matched wild-type (WT) and C/EBP-δ null mice, and analyzed Gr-1+CD11b+ cells by flow cytometry. We did not see any significant difference regarding this population of cells between the two groups of mice (Figure 1C), which indicates no major role of C/EBP-δ in the development of Gr-1+CD11b+ cells in normal development.

Next, we implanted murine lung cancer cells (3LL) into WT and C/EBP-δ null mice for 3 weeks, followed by analysis of MDSCs in spleens, a production site for these cells. As expected, implantation of tumor cells in WT mice resulted in a significant expansion of MDSCs in the spleen (Figure 1D and 1E). Interestingly, loss of C/EBP-δ in mice significantly inhibited tumor-induced expansion of MDSCs (Figure 1D and 1E). Similarly, there was a significant reduction of MDSCs in peripheral blood of C/EBP-δ null tumor bearing mice when compared to corresponding WT controls (Figure 1F). These data support a specific and positive role of C/EBP-δ in the expansion of MDSCs under tumor conditions. C/EBP-δ plays no major role in physiological development of Gr-1+CD11b+ cells.

### Genetic deletion of C/EBP-δ in mice results in fewer myeloid cell infiltration, retarded tumor angiogenesis and tumor growth

**Figure 2:**
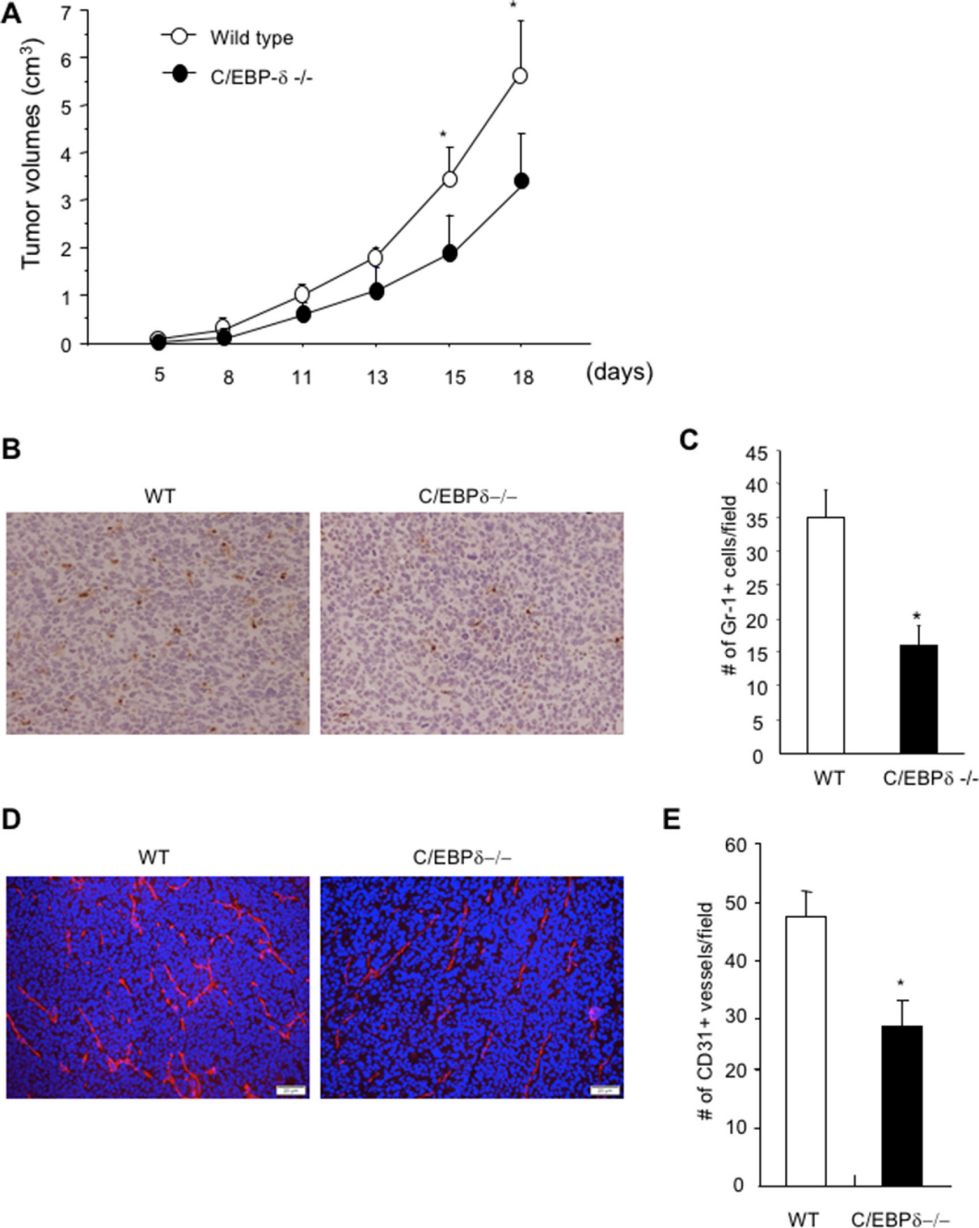
Genetic deletion of C/EBP-δ in mice results in a significant reduction of myeloid cell infiltration, tumor angiogenesis and tumor growth 5 × 10^5^ 3LL tumor cells were s.c. implanted into age and sex matched C/EBP-δ null mice and littermate WT mice. Tumor size was measured, calculated and plotted (Panel **A**). Tumor tissues were harvested from mice with similar size tumors and sectioned. Tissue sections were stained with a Gr-1 specific antibody and counterstained with hematoxylin. Brown color cells are Gr-1 positive cells (Panel **B**). Gr-1+ cells were counted in 10 randomly selected high power fields (Panel **C**). Tumor tissue sections were stained with a CD31 specific antibody (Panel **D**) and CD31+ vessels were counted in 10 randomly selected high power fields (Panel **E**). Data are expressed as mean ± SD. *n* = 10 mice per group, *p < 0.05.

MDSCs are known to infiltrate into tumor tissues, modulate the tumor microenvironment and promote tumor growth and progression [30]. Based on the observation that C/EBP-δ regulates MDSC expansion under tumor conditions, we postulated a pro-tumor role of C/EBP-δ through induction of MDSCs. To test this, we implanted 3LL tumor cells in C/EBP-δ null mice and WT littermates. A measurement of tumor size over a period showed a significant delay in tumor growth in null mice compared with WT mice (Figure 2A). Tumor tissues were harvested from mice with similar size tumors. Tumor sections were stained with an antibody against Gr-1 to mark tumor-infiltrating MDSCs (Figure 2B). There were significant fewer MDSCs in tumor tissues harvested from C/EBP-δ null mice than the ones in WT littermates (Figure 2C). Consistent with the function of MDSC in promoting tumor angiogenesis [30], an analysis on tumor sections confirmed a significant reduction of tumor vascular density associated with C/EBP-δ null conditions compared to controls (Figure 2D and 2E).

**Figure 3:**
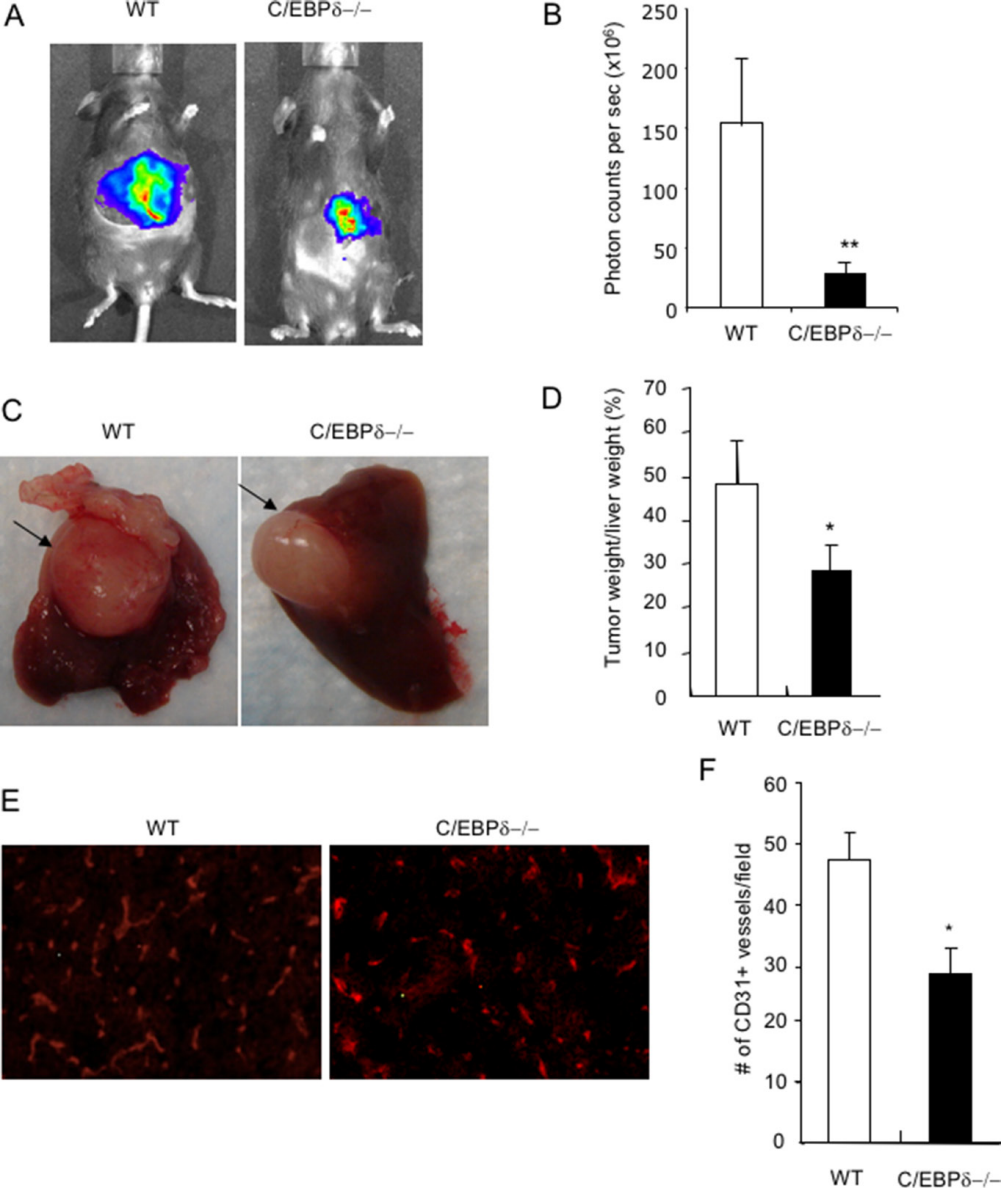
Genetic deletion of C/EBP-δ in mice impairs tumor growth in a liver model Small pieces of MC36-Luc tumor (1 mm^3^) were surgically implanted, one piece in each liver, in age and sex matched C/EBP-δ null mice and littermate WT mice. Tumors were imaged with bioluminescent imaging, and photon counts were used to measure tumor volume 3 weeks after tumor implantation (Panel **A** and **B**). Livers were harvested and grossly examined. Arrows pointed to the tumor (Panel **C**). Tumors were dissected and weighed. The percentage of tumor weight versus the weight of liver was plotted (Panel **D**). Tumor sections were stained with an anti CD31 antibody (Panel **E**), the number of CD31 positive vessels was counted in 10 randomly selected high power fields under microscopy (Panel **F**). Representative images are shown in each study. *n* = 7 mice per group and repeated once, **p < 0.01, *p < 0.05.

To further substantiate the findings and make sure the findings are not limited to one tumor type, we used an orthotopic colorectal tumor model. We implanted a small piece (1 mm^3^) of MC36-Luc colorectal cancer into livers of WT littermates and C/EBP-δ null mice, in which it would develop into a single tumor nodule. This tumor line was stably transfected with a luciferase expression vector that allowed us to measure tumor mass in live mice using molecular imaging. Three weeks after tumor implantation, we measured the tumor mass using bioluminescent imaging in live mice. Consistent with the findings in the 3LL tumor model, the tumor mass measured by photon counts is significantly smaller in the null mice compared to WT littermate controls (Figure 3A and 3B). Tumor tissues were harvested, and gross examination of tumor nodules in the liver and measurement of tumor weight confirmed that there were smaller tumors in the C/EBP-δ null mice than controls (Figure 3C and 3D). Immunofluorescent analysis of tumor sections showed fewer blood vessels in tumors from C/EBP-δ null mice than those from WT mice (Figure 3E and 3F). Based on the pro tumor angiogenesis functions of MDSCs, our data suggest that inactivation of C/EBP-δ impairs the expansion and tumor infiltration of MDSCs, thereby leading to retarded tumor angiogenesis and tumor growth.

**Figure 4:**
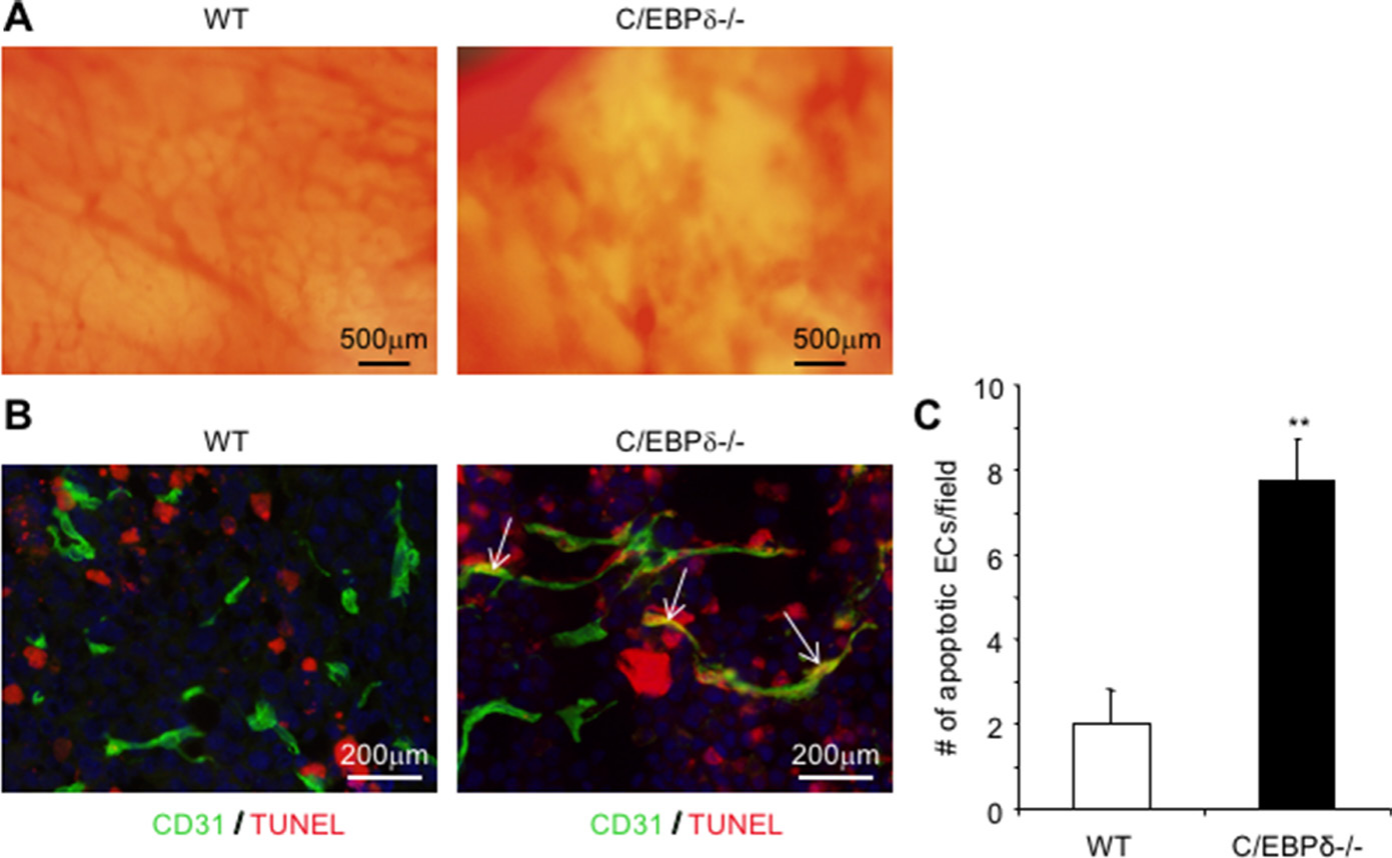
Loss of C/EBP-δ results in increased endothelial apoptosis and hemorrhagic vascular morphology in tumors Dorsal skin vascular window chambers were established in WT and C/EBP-δ null mice, and 1 × 10^5^ 3LL tumor cells were injected into each window chamber. Tumor angiogenesis was imaged in live mice under microscopy 10 days after tumor cell implantation (Panel **A**). Tumor were harvested, sectioned and co-stained with an anti CD31 antibody (green) and TUNEL assay (red) (Panel **B**). The number of double positive cells was counted in 10 randomly selected high power fields under microscopy (Panel **C**). Representative images are shown. *n* = 5 mice per group. **p < 0.01.

Moreover, we implanted 3LL tumor cells into a dorsal vascular window chamber where tumor angiogenesis was directly visualized in live mice [16]. Implantation of tumor cells in WT mice induced a robust tumor angiogenesis. In contrast, there were visibly fewer tumor blood vessels in C/EBP-δ null mice than WT controls (Figure 4A). Notably, tumor vascular structures in the C/EBP-δ null mice display hemorrhagic morphology (Figure 4A). Tumors were harvested from the window model 10 days later. Tumor sections was incubated with an antibody against CD31, an endothelial marker, and co-stained with TUNEL assay. It is quite evident that there were more apoptotic endothelial cells measured as CD31 and TUNEL double positive cells in tumor grown in C/EBP-δ null mice than in WT mice (Figure 4B and 4C). These results provide additional evidence supporting a role of C/EBP-δ in angiogenesis as well as vascular survival in tumor conditions.

### C/EBP-δ is expressed in vascular endothelial cells, and specifically regulates VEGFR2 expression and angiogenesis

**Figure 5:**
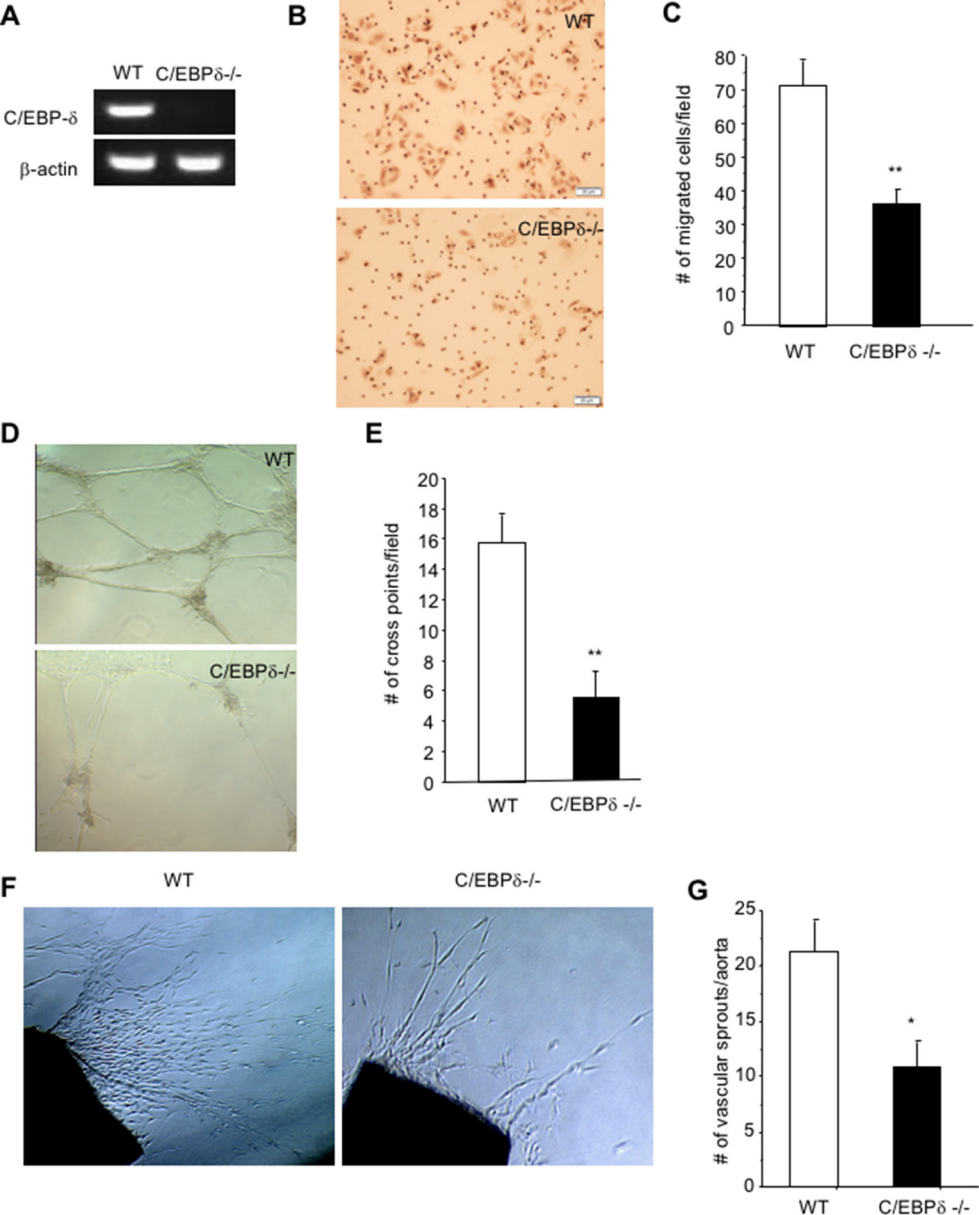
C/EBP-δ is expressed in vascular endothelial cells, and regulates cell motility and angiogenesis Pulmonary microvascular endothelial cells were isolated from age and sex matched C/EBP-δ null mice and littermate WT mice (pooled from 5 mice per group). Expression of C/EBP-δ in endothelial cells was examined by semi quantitative RT-PCR (Panel **A**). Endothelial cell migration was evaluated in a Transwell assay with seeding the primary murine endothelial cells in the upper chamber and addition of recombinant VEGF protein at 20 ng/ml in the bottom chamber (Panel **B**). The migrated cells were counted 5 hours later (Panel **C**). **p < 0.01. Murine endothelial cells were seeded on the top of Matrigel and incubated for 24 hours. Vascular network formation was imaged under microscopy (Panel **D**). The number of cross point of vascular structures was counted in 10 randomly selected fields under microscopy (Panel **E**). **p < 0.01. Aortas collected from age and sex matched WT and C/EBP-δ null mice (*n* = 3 mice per group) were cut into small pieces, embedded in fibrin gel and overlaid with EGM. The number of vascular sprouts was imaged and counted under microscopy after one week in culture (Panel **F** and **G**). **p < 0.01, *p < 0.05. All *in vitro* and *ex vivo* data were done in triplicates and collected from three independent experiments, and expressed as mean ± SD. Representative images are shown in each study.

Impaired tumor angiogenesis and hemorrhagic vascular morphology in C/EBP-δ null mice prompted us to further explore if the gene also directly regulates endothelial function. To this end, we first determined whether C/EBP-δ is present in vascular endothelial cells. We isolated pulmonary microvascular endothelial cells from sex and age matched WT and C/EBP-δ null mice and purified the cells using CD31 magnetic beads (pooled from 5 mice per group). The purity of the cells was confirmed with flow cytometry for CD31 expression and cells with more than 96% purity were used in subsequent studies, which include analyzing the expression of C/EBP-δ by semi quantitative RT-PCR. Indeed, C/EBP-δ is present in vascular endothelial cells isolated from WT mice but absent from cells from the C/EBP-δ null mice (Figure 5A).

Angiogenesis is a multistep process that includes endothelial cell migration, assembly into vascular structures and vascular sprouting. To explore the role of endothelial C/EBP-δ in angiogenesis, we used *in vitro* angiogenic assays in combination with purified pulmonary endothelial cells from mice. A Transwell assay was used to measure endothelial cell migration with seeding the cells in the upper chamber and addition of recombinant VEGF protein in the bottom chamber. Interestingly, loss of C/EBP-δ in endothelial cells significantly impaired cell motility in response to VEGF stimulation compared to WT cells (Figure 5B and 5C). Endothelial cells have the ability to assemble into vascular structures when maintained in a 3-D culture. Consistently, C/EBP-δ null endothelial cells displayed reduced ability to form vascular networks in a Matrigel assay (Figure 5D and 5E). These results reveal a new function of C/EBP-δ in endothelial biology. C/EBP-δ directly regulates angiogenesis through an effect on endothelial cell motility and vascular assembly. This conclusion is further supported by a more complex *ex vivo* aortic ring assay. Endothelial sprouts spontaneously develop from a small aortic tissue when maintained in a 3-D culture. As expected, C/EBP-δ null aortic tissue developed significantly fewer vascular sprouts than the WT control (Figure 5F and 5G). Thus, these data confirm a positive and direct role of endothelial C/EBP-δ in angiogenesis.

**Figure 6:**
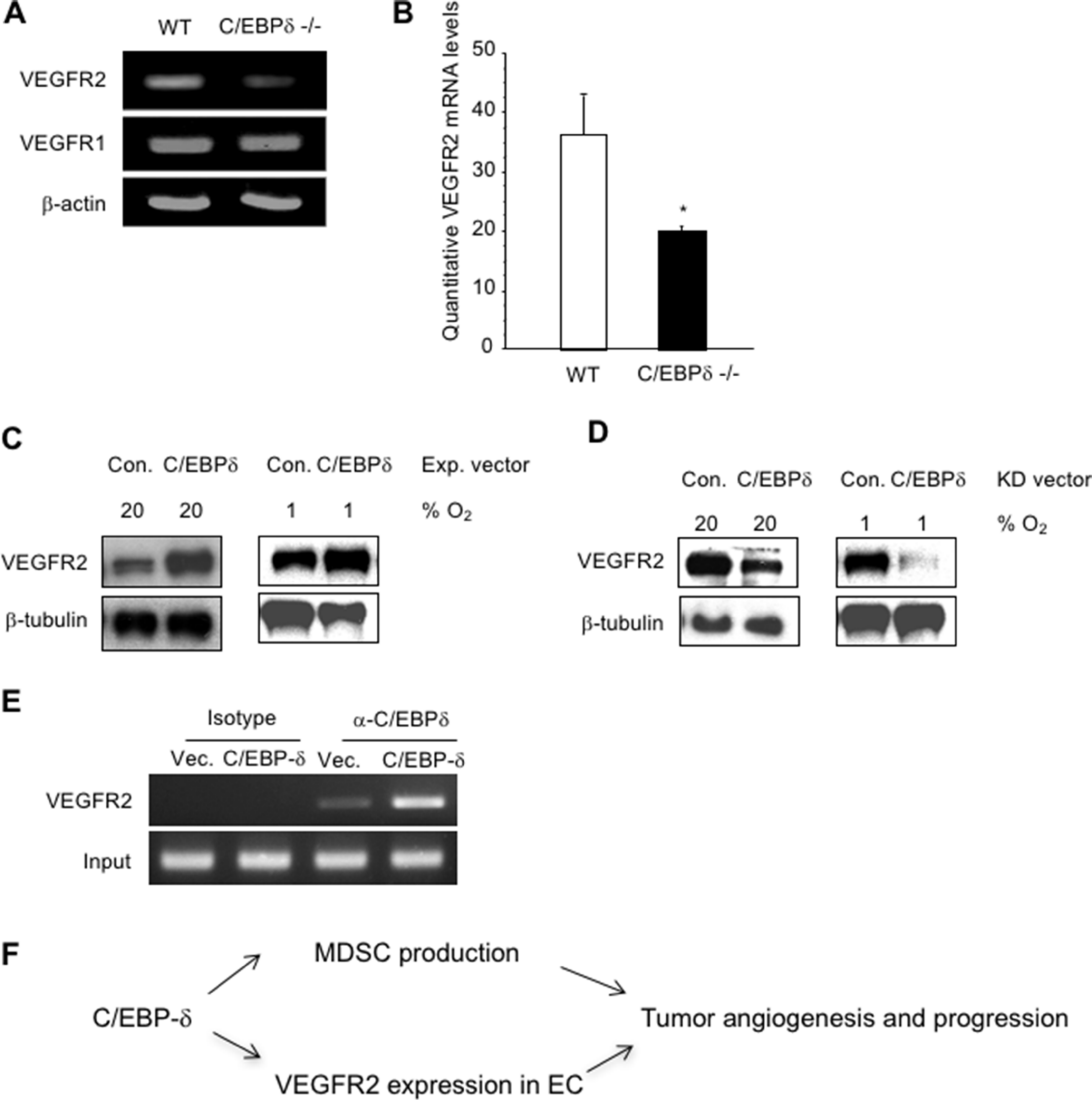
C/EBP-δ binds to the promoter region of VEGFR2 and regulates its expression in endothelial cells The mRNA levels of VEGFR1 in murine endothelial cells from WT and C/EBP-δ null mice were analyzed by semi quantitative RT-PCR (Panel **A**), and the mRNA levels of VEGFR2 were measured by both semi quantitative RT-PCR (Panel A) and qPCR (Panel **B**). *p < 0.05. HUVECs were transfected with either empty vector or C/EBP-δ expression vector for 24 hours. The cells were then cultured under either normoxia (20% O_2_) or hypoxia (1% O_2_) for another 24 hours. VEGFR2 protein levels were measured by Western blot (Panel **C**). HUVECs were transfected with either control shRNA or shRNA for C/EBP-δ for 24 hours. The cells were then cultured under either normoxia or hypoxia for another 24 hours. VEGFR2 protein levels were measured by Western blot (Panel **D**). HUVECs were transfected with either control vector or C/EBP-δ expression vector for 70 hours. ChIP assay was performed and the VEGFR2 promoter region was amplified by PCR (Panel **E**). Each experiment was repeated three times. Representative images are shown. Diagram of C/EBP-δ mediated tumor angiogenesis and MDSC (Panel **F**)expansion in tumor progression (Panel **F**).

Since deletion of C/EBP-δ in endothelial cells inhibited its response to VEGF stimulation, we therefore analyzed VEGF receptor expression on the cells. Very interestingly, there was a significant reduction in VEGFR2, but not VEGFR1, in the null endothelial cells compared to WT cells (Figure 6A and 6B). To confirm a role of C/EBP-δ in VEGFR2 expression in endothelial cells, we used human umbilical vein endothelial cells (HUVECs). We found that ectopic expression of C/EBP-δ in endothelial cells increased the protein expression of VEGFR2, and hypoxia seems have no major impact on this gene induction (Figure 6C). Conversely, knockdown of C/EBP-δ in endothelial cells inhibited VEGFR2 protein expression, which is independent of hypoxia (Figure 6D). These results reveal a novel role of C/EBP-δ in the regulation of VEGFR2 expression in endothelium, which provides molecular evidence linking loss of C/EBP-δ with defective angiogenesis and hemorrhagic vascular morphology observed in tumor studies. On the other hand, we did not see a difference in VEGFR2 expression between hypoxic and normoxic conditions in either condition, suggesting a HIF independent mechanism. This finding is in line with publications that hypoxia does not regulate VEGFR2 expression in endothelial cells [6].

Finally, we investigated the gene regulation mechanism. Using a ChIP assay, we found that C/EBP-δ was recruited to the VEGFR2 promoter region, which contains a C/EBP binding site at -596 to -582 and conserved between mice and humans. There is a faint binding in empty vector transfected cells indicative of recruitment of endogenous C/EBP-δ protein to the VEGFR2 promoter (Figure 6E). Collectively, these data reveal a new function of C/EBP-δ in endothelial biology. C/EBP-δ is present in vascular endothelial cells and regulates VEGFR2 expression, likely at the transcription level. Loss of C/EBP-δ reduces VEGFR2 expression in endothelium, which contributes to defective angiogenesis and increased endothelial apoptosis in tumors associated with C/EBP-δ null conditions.

## DISCUSSION

Myeloid cells and vascular endothelial cells are two major components that constitute the tumor microenvironment. These cells create a permissive microenvironment that enables tumor growth, progression and metastasis. In this study, we have identified a common regulator in C/EBP-δ that positively regulates MDSC expansion and VEGFR2 expression in vascular endothelium. C/EBP-δ is elevated in tumor derived MDSCs, and genetic deletion of the gene in mice specifically inhibits tumor induced expansion of MDSCs, yet loss of the gene has no major effect on normal Gr-1+CD11b+ cell development. Consequently, tumor growth is retarded in the null mice compared to WT controls, which is associated with a significant reduction of MDSC infiltration in tumors and tumor angiogenesis. These changes are in line with the properties of MDSCs that infiltrate into tumors, modulate the tumor microenvironment and promotes tumor angiogenesis [30]. Moreover, loss of C/EBP-δ causes a significant increase of endothelial apoptosis in tumors. Further studies reveal expression of C/EBP-δ in vascular endothelial cells, and C/EBP-δ directly regulated endothelial motility and angiogenesis. Notably, inactivation of C/EBP-δ in endothelial cells specifically inhibits the expression of VEGFR2, but not VEGFR1, which likely occurred at the transcription level. Collectively, these findings reveal dual positive roles of C/EBP-δ in tumorigenesis through its effects on MDSC expansion as well as VEGFR2 expression in endothelial cells, which together promote tumor angiogenesis and tumor growth (Figure 6F).

C/EBP-δ belongs to the C/EBP transcription family, and is present in a variety of cells and tissues with low to undetectable levels in normal situations. Consistently, in this study we did not find major differences in MDSC expansion and angiogenesis in normal development regarding the status of C/EBP-δ. C/EBP-δ is rapidly induced by a variety of stimuli, including inflammatory cytokines [23]. Recently, it was reported that hypoxia upregulates C/EBP-δ expression in murine mammary tumor cells. The authors show that C/EBP-δ enhances mTOR/Akt/S6K1 signaling and augments translation and activity of HIF-1α [1]. As inflammation and hypoxia are two conditions closely associated with tumor development, it is no surprise that we observe a strong induction of C/EBP-δ expression in MDSCs by tumor conditions. Consequently, elevated C/EBP-δ induces MDSC expansion in myeloid cells as well as VEGFR2 expression in vascular endothelium, which together promote tumor angiogenesis and tumor progression.

A major function of C/EBPs is to regulate cell differentiation, which includes myeloid cells. C/EBP-δ is expressed in myeloid lineage cells, and its levels change during cell differentiation [3, 24]. Inactivation of C/EBP-δ in mice causes incomplete maturation of granulocytes with a lack of specific granule proteins [29]. In this study, we did not observe a major change of Gr-1+CD11b+ cells when C/EBP-δ is deleted in mice. However, loss of the gene significantly inhibits the production of MDSCs under tumor conditions. These data implicate a specific role of C/EBP-δ in emergency myelopoiesis under pathological conditions. Consistent with our findings on C/EBP-δ, it was reported that hematopoietic deletion of C/EBPβ, another member of the C/EBPs, led to a decrease in the number of MDSCs and a loss in the tolerance promoting activities of MDSCs, accompanied by a difference in differentiation of myeloid cells [17]. Our data, along with published findings, point to important roles for this family of transcription factors in MDSC biology.

Another important function of C/EBP transcription factors is to regulate gene expression, such as VEGFR3 in lymphatic endothelial cells [21], platelet-derived growth factor receptor in vascular smooth muscle cells [7, 14, 32], insulin-like growth factor-I in osteoblast cells [4, 11], and CXCR4 in tumor cells [1]. Our current study adds another important target gene of C/EBP-δ in vascular endothelial cells. We show for the first time that C/EBP-δ is expressed in vascular endothelial cells, and that specifically regulates the expression of VEGFR2 but not VEGFR1. C/EBP-δ is recruited to the promoter region of VEGFR2 gene, indicative transcriptional regulation. The angiogenic functions of VEGF are mediated through cell surface receptors on endothelial cells. Among them, VEGFR2 mediates the majority of the downstream effects of VEGF in angiogenesis and vascular survival. Based on these, it is conceivable to suggest that loss of C/EBP-δ associated inhibition of tumor angiogenesis and increased endothelial apoptosis in tumors is partly due to a reduction of VEGFR2 expression in vascular endothelium.

In summary, this study reveals a common mediator in C/EBP-δ that regulates both MDSC expansion and VEGFR2 mediated angiogenesis, two processes critical for tumor development. C/EBP-δ is elevated in tumor derived MDSCs. Importantly, loss of the C/EBP-δ in mice significantly impaired tumor-induced expansion of MDSCs, and tumor angiogenesis as well as vascular survival. Notably, loss of C/EBP-δ has no major effect on angiogenesis and Gr-1+CD11b+ myeloid cell differentiation during normal development. These data implicate a specific role of C/EBP-δ in pathological development. Induction of C/EBP-δ by tumor conditions creates a permissive microenvironment for tumor growth and progression. Thus, targeting C/EBP-δ may provide an interesting avenue for cancer therapy; through which one could simultaneously target MDSCs and tumor endothelium, two important components in the tumor microenvironment, to achieve killing two birds with one stone.

## MATERIALS AND METHODS

### Mice and tumor models

The mice were maintained in a pathogen-free facility at Vanderbilt University and the National Cancer Institute (Frederick, MD) in accordance with Animal Care and Use Committee regulations. C/EBP-δ null mice in the C57BL/6 background were kindly provided by Dr. Sterneck at the NCI [26]. Heterozygous C/EBP-δ mice were mated to generate null mice and WT littermate controls. 3LL murine lung cancer models and a luciferase stably transfected murine colorectal cancer line, MC36-Luc, were used in this study. Both tumor lines are syngeneic to C57BL/6 mice. 3LL cells (5 × 10^5^) were injected subcutaneously into the left flank of mice. The size of tumors was determined a caliper. The equation volume = length × (width)^2^ × 0.5 was used to calculate tumor volume. 8 weeks old female mice were used for the tumor vascular window model as described [16].

For liver tumor implantation, six-week-old female mice were anesthetized. A subcostal incision was made, and the left lobe of the liver was exteriorized. A small piece of MC36-Luc tumor (1 mm^3^) harvested from a donor mouse was surgically implanted on the left lateral lobe. A surgical suture was used to fix the tumor piece within the liver tissue. Three weeks later, tumor volume was measured by bioluminescent imaging and photon signal intensities were counted. Tumors were harvested and weighed.

### Flow cytometry

Cell suspensions of spleen and blood samples were prepared, counted, and incubated with PE-CD11b and FITC-Gr-1 antibody conjugates (BD Biosciences), as described [30]. The cells were analyzed on a FACScan flow cytometer (Becton Dickinson, Mountain View, CA).

### Pulmonary vascular endothelial cells and angiogenic assays

Lungs from age and sex matched C/EBP-δ null mice and littermate WT mice (pooled from 6 mice per group) were processed to generate single cell suspension [5]. Vascular endothelial cells purified with biotinylated anti-CD31 (Mec 13.3) magnetic beads (EasySep Biotin Selection Kit; Stem Cell Technologies). The cells were cultured in gelatin-coated flasks and maintained in EGM (Clonetics). Cell purity was confirmed by Flow with an anti-CD31 antibody. Cells used in each study were greater than 96% pure. Standard *in vitro* and *ex vivo* angiogenesis assays were performed as described [5].

### Immunohistochemistry

Tumors were harvested and processed at the end of each experiment. Frozen tissue sections were incubated with CD31 antibody or Gr-1 antibody (BD Pharmingen) as described [30, 31]. Apoptotic cells were evaluated by the ApopTag^®^ Red *In Situ* Apoptosis Detection Kit (Chemicon) per manufacturer's instruction. Vascular density, myeloid infiltration and apoptotic endothelial cells were calculated by counting CD31 positive vessels, Gr-1 positive cells and CD31 and TUNEL double positive cells, respectively, in ten randomly selected high power fields under microscopy.

### Overexpression and knockdown of C/EBP-δ in endothelial cells

HUVECs from passages 3–6 were transfected using HUVEC Nucleofector Kit (Lonza, VPB-1002). Twenty-four hours later, the cells were incubated under either normoxia (20% O_2_) or hypoxia conditions (1% O_2_) for 48 hours followed by analysis of gene expression. For ectopic expression, the cells were transfected with PCDNA 3.1-C/EBP-δ expression plasmid. For knockdown, we used shRNA plasmid DNA specific for C/EBP-δ (Sigma-Aldrich). Non-specific shRNA plasmid was used as a control. Puromycin at 1 μg/ml was used for enrichment of shRNA construct transfected cells prior to each experiment.

### Chromatin immunoprecipitation (ChIP)

HUVECs were transfected with 5 μg of either empty vector or C/EBP-δ plasmid. ChIP was performed using a ChIP-IT Express Kit (Active Motif), and C/EBP-δ antibody and isotype control (M-17X, Santa Cruz). The input and ChIP DNA samples were subjected to PCR. The promoter region of VEGFR2 was analyzed using Genomatix software, and primers were designed around predicted C/EBP site to yield PCR products of approximately 150–250 base pairs.

### Statistical analysis

The results are presented as means ± SD for each sample. The statistical significance of differences was determined by Student's two-tailed *t* test. All data were calculated with a Statview 5.0 (Abacus Concepts, Berkeley, CA) statistical software package run on a Windows computer. The differences were considered statistically significant when *p*-value < 0.05.

## References

[1] Balamurugan K, Wang JM, Tsai HH, Sharan S, Anver M, Leighty R, Sterneck E (2010). The tumour suppressor C/EBPdelta inhibits FBXW7 expression and promotes mammary tumour metastasis. Embo J.

[2] Balamurugan K, Sharan S, Klarmann KD, Zhang Y, Coppola V, Summers GH, Roger T, Morrison DK, Keller JR, Sterneck E (2013). FBXW7alpha attenuates inflammatory signalling by downregulating C/EBPdelta and its target gene Tlr4. Nat Commun.

[3] Bjerregaard MD, Jurlander J, Klausen P, Borregaard N, Cowland JB (2003). The in vivo profile of transcription factors during neutrophil differentiation in human bone marrow. Blood.

[4] Chang W, Parra M, Centrella M, McCarthy TL (2005). Interactions between CCAAT enhancer binding protein delta and estrogen receptor alpha control insulin-like growth factor I (igf1) and estrogen receptor-dependent gene expression in osteoblasts. Gene.

[5] DeBusk LM, Boelte K, Min Y, Lin PC (2010). Heterozygous deficiency of delta-catenin impairs pathological angiogenesis. J Exp Med.

[6] Ferrara N, Gerber HP, LeCouter J (2003). The biology of VEGF and its receptors. Nat Med.

[7] Fukuoka T, Kitami Y, Okura T, Hiwada K (1999). Transcriptional regulation of the platelet-derived growth factor alpha receptor gene via CCAAT/enhancer-binding protein-delta in vascular smooth muscle cells. J Biol Chem.

[8] Gabrilovich DI, Nagaraj S (2009). Myeloid-derived suppressor cells as regulators of the immune system. Nat Rev Immunol.

[9] Gery S, Tanosaki S, Hofmann WK, Koppel A, Koeffler HP (2005). C/EBPdelta expression in a BCR-ABL-positive cell line induces growth arrest and myeloid differentiation. Oncogene.

[10] Ishii Y, Kasukabe T, Honma Y (2005). Induction of CCAAT/enhancer binding protein-delta by cytokinins, but not by retinoic acid, during granulocytic differentiation of human myeloid leukaemia cells. Br J Haematol.

[11] Ji C, Chang W, Centrella M, McCarthy TL (2003). Activation domains of CCAAT enhancer binding protein delta: regions required for native activity and prostaglandin E2-dependent transactivation of insulin-like growth factor I gene expression in rat osteoblasts. Mol Endocrinol.

[12] Johnson PF (2005). Molecular stop signs: regulation of cell-cycle arrest by C/EBP transcription factors. J Cell Sci.

[13] Kim Y, Fischer SM (1998). Transcriptional regulation of cyclooxygenase-2 in mouse skin carcinoma cells. Regulatory role of CCAAT/enhancer-binding proteins in the differential expression of cyclooxygenase-2 in normal and neoplastic tissues. J Biol Chem.

[14] Kitami Y, Fukuoka T, Hiwada K, Inagami T (1999). A high level of CCAAT-enhancer binding protein-delta expression is a major determinant for markedly elevated differential gene expression of the platelet-derived growth factor-alpha receptor in vascular smooth muscle cells of genetically hypertensive rats. Circ Res.

[15] Lekstrom-Himes J, Xanthopoulos KG (1998). Biological role of the CCAAT/enhancer-binding protein family of transcription factors. J Biol Chem.

[16] Lin P, Polverini P, Dewhirst M, Shan S, Rao PS, Peters K (1997). Inhibition of tumor angiogenesis using a soluble receptor establishes a role for Tie2 in pathologic vascular growth. J Clin Invest.

[17] Marigo I, Bosio E, Solito S, Mesa C, Fernandez A, Dolcetti L, Ugel S, Sonda N, Bicciato S, Falisi E, Calabrese F, Basso G, Zanovello P (2010). Tumor-induced tolerance and immune suppression depend on the C/EBPbeta transcription factor. Immunity.

[18] Marx J (2008). Cancer immunology. Cancer’s bulwark against immune attack: MDS cells. Science.

[19] Marx J (2008). Cancer biology. All in the stroma: cancer's Cosa Nostra. Science.

[20] Milde-Langosch K, Loning T, Bamberger AM (2003). Expression of the CCAAT/enhancer-binding proteins C/EBPalpha, C/EBPbeta and C/EBPdelta in breast cancer: correlations with clinicopathologic parameters and cell-cycle regulatory proteins. Breast Cancer Res Treat.

[21] Min Y, Ghose S, Boelte K, Li J, Yang L, Lin PC (2011). C/EBP-regulates VEGF-C autocrine signaling in lymphangiogenesis and metastasis of lung cancer through HIF-1alpha. Oncogene.

[22] Rabek JP, Scott S, Hsieh CC, Reisner PD, Papaconstantinou J (1998). Regulation of LPS-mediated induction of C/EBP delta gene expression in livers of young and aged mice. Biochim Biophys Acta.

[23] Ramji DP, Foka P (2002). CCAAT/enhancer-binding proteins: structure, function and regulation. Biochem J.

[24] Scott LM, Civin CI, Rorth P, Friedman AD (1992). A novel temporal expression pattern of three C/EBP family members in differentiating myelomonocytic cells. Blood.

[25] Shojaei F, Wu X, Malik AK, Zhong C, Baldwin ME, Schanz S, Fuh G, Gerber HP, Ferrara N (2007). Tumor refractoriness to anti-VEGF treatment is mediated by CD11b+Gr1+ myeloid cells. Nat Biotechnol.

[26] Sterneck E, Paylor R, Jackson-Lewis V, Libbey M, Przedborski S, Tessarollo L, Crawley JN, Johnson PF (1998). Selectively enhanced contextual fear conditioning in mice lacking the transcriptional regulator CCAAT/enhancer binding protein delta. Proc Natl Acad Sci USA.

[27] Takata Y, Kitami Y, Yang ZH, Nakamura M, Okura T, Hiwada K (2002). Vascular inflammation is negatively autoregulated by interaction between CCAAT/enhancer-binding protein-delta and peroxisome proliferator-activated receptor-gamma. Circ Res.

[28] Tanaka T, Yoshida N, Kishimoto T, Akira S (1997). Defective adipocyte differentiation in mice lacking the C/EBPbeta and/or C/EBPdelta gene. Embo J.

[29] Yamanaka R, Lekstrom-Himes J, Barlow C, Wynshaw-Boris A, Xanthopoulos KG (1998). CCAAT/enhancer binding proteins are critical components of the transcriptional regulation of hematopoiesis (Review). Int J Mol Med.

[30] Yang L, DeBusk LM, Fukuda K, Fingleton B, Green-Jarvis B, Shyr Y, Matrisian LM, Carbone DP, Lin PC (2004). Expansion of myeloid immune suppressor Gr+CD11b+ cells in tumor-bearing host directly promotes tumor angiogenesis. Cancer Cell.

[31] Yang L, Huang J, Ren X, Gorska AE, Chytil A, Aakre M, Carbone DP, Matrisian LM, Richmond A, Lin PC, Moses HL (2008). Abrogation of TGF beta signaling in mammary carcinomas recruits Gr-1+CD11b+ myeloid cells that promote metastasis. Cancer Cell.

[32] Yang ZH, Kitami Y, Takata Y, Okura T, Hiwada K (2001). Targeted overexpression of CCAAT/enhancer-binding protein-delta evokes enhanced gene transcription of platelet-derived growth factor alpha-receptor in vascular smooth muscle cells. Circ Res.

[33] Zhang DE, Hetherington CJ, Meyers S, Rhoades KL, Larson CJ, Chen HM, Hiebert SW, Tenen DG (1996). CCAAT enhancer-binding protein (C/EBP) and AML1 (CBF alpha2) synergistically activate the macrophage colony-stimulating factor receptor promoter. Mol Cell Biol.

